# Association between cognitive performance and self-reported glaucoma in middle-aged and older adults: a cross-sectional analysis of ELSA-Brasil

**DOI:** 10.1590/1414-431X202010347

**Published:** 2020-10-30

**Authors:** K.S. Vidal, C.K. Suemoto, A.B. Moreno, B. Duncan, M.I. Schmidt, M. Maestri, S.M. Barreto, P.A. Lotufo, L. Bertola, I.M. Bensenor, A.R. Brunoni

**Affiliations:** 1 Laboratório da Visão, Instituto de Psicologia, Universidade de São Paulo, São Paulo, SP, Brasil; 2 Divisão de Geriatria, Faculdade de Medicina, Universidade de São Paulo, São Paulo, SP, Brasil; 3 Departamento de Epidemiologia e Métodos Quantitativos na Saúde, Escola Nacional de Saúde Pública, Fundação Oswaldo Cruz, Rio de Janeiro, RJ, Brasil; 4 Departamento de Epidemiologia, Universidade Federal do Rio Grande do Sul, Porto Alegre, RS, Brasil; 5 Departamento de Oftalmologia e Otorrinolaringologia, Faculdade de Medicina, Universidade do Rio Grande do Sul, Porto Alegre, RS, Brasil; 6 Faculdade de Medicina e Hospital das Clínicas, Universidade Federal de Minas Gerais, Belo Horizonte, MG, Brasil; 7 Departamento de Clínica Médica, Hospital Universitário, Universidade de São Paulo, São Paulo, SP, Brasil; 8 Departamento de Medicina Interna, Faculdade de Medicina, Universidade de São Paulo, São Paulo, SP, Brasil; 9 Laboratório de Neurociências, Departamento e Instituto de Psiquiatria, Faculdade de Medicina, Universidade de São Paulo, São Paulo, SP, Brasil

**Keywords:** Cohort study, Cognitive performance, Glaucoma, Retinal diseases, Elderly

## Abstract

Recent evidence suggests that glaucoma and Alzheimer's disease are neurodegenerative diseases sharing common pathophysiological and etiological features, although findings are inconclusive. We sought to investigate whether self-reported glaucoma patients without dementia present poorer cognitive performance, an issue that has been less investigated. We employed cross-sectional data from the Brazilian Longitudinal Study of Adult Health (ELSA-Brasil) and included participants ≥50 years of age without a known diagnosis of dementia and a self-reported glaucoma diagnosis. We excluded those with previous stroke, other eye conditions, and using drugs that could impair cognition. We evaluated cognition using delayed word recall, phonemic verbal fluency, and trail making (version B) tests. We used multinomial linear regression models to investigate associations between self-reported glaucoma with cognition, adjusted by several sociodemographic and clinical variables. Out of 4,331 participants, 139 reported glaucoma. Fully-adjusted models showed that self-reported glaucoma patients presented poorer performance in the verbal fluency test (β=-0.39, 95%CI=-0.64 to -0.14, P=0.002), but not in the other cognitive assessments. Thus, our results support the hypothesis that self-reported glaucoma is associated with poor cognitive performance; however, longitudinal data are necessary to corroborate our findings.

## Introduction

Glaucoma is one of the leading causes of preventable blindness worldwide (1). It is an eye disease characterized by progressive damage to the optic nerve head and retinal nerve fiber layer (RNFL), and may be associated with elevated intraocular pressure in most cases, leading to increasing vision loss and, eventually, blindness (2). Accumulating evidence suggests an association between glaucoma and Alzheimer's disease (AD), as both conditions share similar epidemiology, such as increased prevalence in older ages (3), impairment of magnocellular visual processing (4), and pathophysiological features. For instance, AD-related neuropathological findings, such as vacuolar degeneration, neuronal apoptosis, neurofibrillary tangles, and amyloid-B plaques, have been observed in the retina as well (3,5), while abnormal retinal findings have been observed in AD patients, such as reduction of retinal ganglion cells (RGCs) (6), RNFL (7), and macular thickness (8). Moreover, cohort studies showed that glaucoma patients have an increased risk for dementia (9), and dementia patients have an increased prevalence of glaucoma (10).

However, cognitive performance in middle-aged individuals with glaucoma (without dementia) has been relatively less investigated, which could provide useful insights for the missing links on the actual mechanisms contributing to the association between glaucoma and dementia. A large study with 1,485 healthy, middle-aged individuals showed an association between RNFL and better cognitive performance (11). This finding is relevant since in glaucoma progression, the RNFL becomes progressively thinner. Notwithstanding, in one cohort study of 839 participants ≥85 years old, no convincing evidence for an association between mini-mental state examination (MMSE) and glaucoma was found (12). In addition, a study with 137 participants found that patients with glaucoma presented lower performance in the Montreal Cognitive Assessment (MoCA) compared to controls, but only after adjustment for confounding variables (13). In contrast, a small study with 60 participants showed lower MMSE scores in glaucoma *vs* controls (14). These different findings could be explained by several limitations in study design, including improper handling of confounding variables (such as age, educational level, ethnicity, and clinical comorbidities) and inadequate selection of outcome variables, as MMSE and MoCA are primarily cognitive screening questionnaires, being suboptimal for the assessment of specific cognitive functions. Moreover, most studies were not conducted in low- and middle-income countries that present distinct characteristics regarding ethnicity, socioeconomic conditions, and clinical and neuropsychiatric comorbidities.

Considering these issues, we investigated cognitive performance in patients with self-reported glaucoma using cross-sectional data from a large Brazilian cohort, the Brazilian Longitudinal Study of Adult Health (ELSA-Brasil). According to the assumption that glaucoma is a neurodegenerative illness that might not only affect the visual system (3), we hypothesized that these patients would present lower cognitive performance in the evaluated domains of ELSA-Brasil's cognitive assessment.

## Material and Methods

### Overview of ELSA-Brasil

ELSA-Brasil is a cohort of civil servants based on six universities and research institutions in major Brazilian cities (São Paulo, Rio de Janeiro, Salvador, Porto Alegre, Belo Horizonte, and Vitoria) (15). The study was approved by the local ethics committees, and all participants provided written, informed consent prior to entering into the study. The first wave (baseline, n=15,105 participants) of ELSA-Brasil took place from August 2008 to December 2010. Eligible subjects included all active or retired employees of these institutions, who were between the ages of 35 and 74 years old, and free of dementia at enrollment. Exclusion criteria at baseline were current or recent (<4 months to the first interview) pregnancy, intention to quit working at the institution soon, severe cognitive or communication impairments that hindered an interview, and, if retired, residence outside the metropolitan area of the corresponding study center.

Data from participants were collected using a structured questionnaire about sociodemographic conditions and self-reported health and disease history, and evaluation of prevalent classical cardiovascular risk factors, clinical variables, laboratory examination via venipuncture, anthropometric measurements, blood pressure, and use of medications according to the Anatomic Therapeutic Chemical (ATC) code. Measurements followed standardized protocols and were collected by certified and trained technicians.

### Study population 

For the present analysis, we included only participants who had: 1) data on self-reported diagnosis of glaucoma; 2) complete data of exposure, outcomes, or covariates; and 3) ≥50 years old, as the prevalence of glaucoma and cognitive deficits in younger adults is very low. We excluded subjects presenting confounding variables such as self-reported diagnosis of 1) previous stroke, 2) other self-reported eye conditions, such as cataracts, retinal diseases such as diabetic retinopathy, age-related macular degeneration, and 3) using drugs that could interfere with cognitive function such as neuroleptics, antiparkinsonian drugs, anticonvulsants, and benzodiazepines.

**Figure 1:**
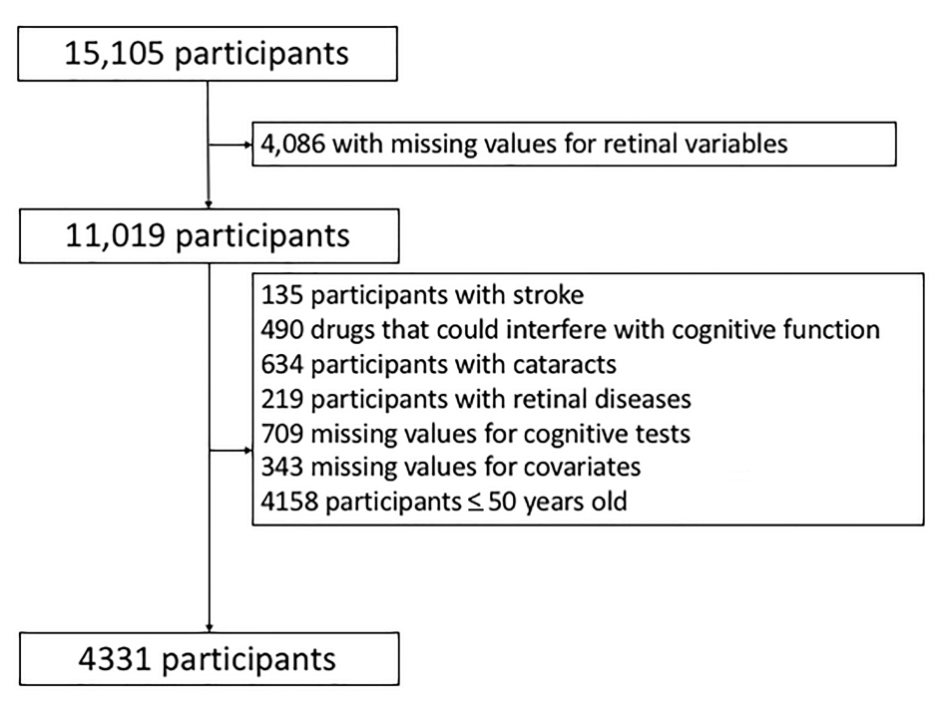
Study flow chart.

Therefore, for the present study, out of 15,105 participants, 4,086 subjects presented missing values for eye variables and 4,158 were less than 50 years old. Other reasons for non-inclusion are described in Figure 1. Therefore, data from 4,331 participants were analyzed.

### Assessments

#### Eye assessments

Self-reported eye diseases (glaucoma, cataracts, macular degeneration, corneal diseases, dry eye, age-related macular retinopathy degeneration, and diabetic retinopathy), history of use of eye drops, ointments, lubricants, gels, and medications, and of eye surgeries and interventions were assessed using a standardized questionnaire. If “Yes” was the answer given to the initial question about previous glaucoma diagnosis, participants had to answer complementary questions regarding “previous glaucoma surgery”, “use of pilocarpine eye drops”, “use of [other] eye drop medications” (such as beta-blockers, prostaglandin analogues, and others). In addition, participants were asked to classify the quality of their vision without correction on a 5-point scale (Excellent, Good, Regular, Bad, and Very Bad).

#### Assessments of Cognitive Function

Cognitive function was assessed using the following tests: a) the Consortium to Establish a Registry for Alzheimer's Disease Word List Memory Test (CERAD-WLMT) (16); b) the Phonemic Verbal Fluency Test (PVFT) (17); and c) the Trail Making Test version B (TMT) (18). Trained examiners administered these tests in a fixed order during a single session, in a quiet room with good lighting and low levels of noise or other distractions. We used the Brazilian version of the CERAD-WLMT (19). This test comprises immediate word recall, delayed word recall, and word recognition, and evaluates the memory domain (16). Participants were asked to read and learn 10 words after three exposures; the sum of the number of words recalled in each of these attempts was the score in the immediate word recall. After a 5-min filled delay, the participants were given 60 s to recall the words. The delayed word recall score was equal to the number of recalled words. Finally, 20 words were presented and the participants had to recognize the 10 words that were presented previously. In the PVFT, participants were asked to generate as many words as possible that started with the letter F to evaluate language and executive ability. The score on this test was the total number of generated words (20). Finally, the TMT is a test of executive function, processing speed, and visual-spatial organization. The participants were instructed to draw lines connecting letters and numbers in an order that alternated between increasing numeric values and alphabetic order. The test score was the total time taken to complete the task, in seconds.

#### Assessment of covariates

Sociodemographic covariates were collected using structured questionnaires (15) and were categorized in: 1) age; 2) sex; 3) ethnicity (white *vs* non-white); and 4) education (having at least a university degree).

Presence of any depressive disorder was also included as a covariate. It was assessed using the validated Brazilian version of the Clinical Interview Schedule-Revised (CIS-R) (21), a structured interview for measurement and diagnosis of non-psychotic psychiatric morbidity in community, applied by trained interviewers, who are as reliable as psychiatrists in using CIS-R for performing mental diagnosis. (22) Introductory questions from the CIS-R about appetite and fluctuations in weight have not been included in the ELSA-Brasil questionnaire at wave 1. As a result, the prevalence of depressive episodes may have been slightly underestimated.

Anthropometric measurements were assessed using standard techniques (23). Blood pressure was measured in a seated position, after 5 min of rest, with a validated oscillometric device (Omron HEM 705CPINT, Japan). Three measurements were performed with 1-min intervals and the mean of the last two readings was considered. Venous blood samples were obtained following an overnight fast. Enzymatic assays of the centrifuged serum - colorimetric and hexokinase method (ADVIA 1200, Siemens^®^, Germany) - determined total cholesterol and fasting plasma glucose, respectively. Clinical risk factors included as covariates were: 1) presence of hypertension (use of antihypertensive drug, systolic blood pressure ≥140 mm Hg or diastolic blood pressure ≥90 mm Hg); 2) presence of diabetes mellitus (self-reported, use of oral hypoglycemic agents or insulin therapy, fasting plasma glucose ≥126 mg/dL, 2-h postprandial 75 g glucose test ≥200 mg/dL, or glycosylated hemoglobin ≥6.5%); 3) ideal cholesterol levels (total cholesterol <200 mg/dL in the absence of lipid-lowering medication); 4) ideal smoking status (no smoking history or quit smoking >2 years *vs* otherwise); 5) ideal physical activity (defined as ≥75 min/week of vigorous physical activity, or ≥150 min/week of moderate physical activity, or a combination of both *vs* otherwise), assessed using the International Physical Activity Questionnaire (24); 6) presence of cardiac conditions, such as coronary artery disease (previous self-reported myocardial infarction or myocardial revascularization), and/or self-reported heart failure; 7) body mass index (measured in kg/m^2^); 8) excessive alcohol use (defined as those with an ethanol consumption ≥210 and ≥140 g/week, for men and women, respectively (25)) and assessed using the Alcohol Use Questionnaire (26); and 9) presence of thyroid dysfunctions, such as subclinical or clinical hypo- or hyperthyroidism (normal range: TSH from 0.4 to 4 mIU/L and FT4 levels from 0.8 to 1.9 ng/dL; hypothyroidism: TSH >4.0 mIU/L and FT4 <0.8 ng/dL; and hyperthyroidism: TSH <0.4 mIU/dL and FT4 >1.9 ng/dL).

### Statistical analyses

Analyses were performed with Stata 16 (Statacorp, USA). We describe sociodemographic and clinical data using frequencies for categorical variables, and mean and standard deviations (SD) for continuous variables. Statistical significance was set under an alpha threshold of 0.005, considering the multiple comparisons performed in our analyses. This lower threshold (compared to the “standard” 0.05) has been proposed as a straightforward method to reduce the rate of false-positive findings (27) and was already used in previous studies from our group (28).

The raw scores on the cognitive tests were transformed into z-scores by subtracting each participant's test score from the mean score of the sample and dividing the difference by the SD of the sample. The z-scores of the TMT were multiplied by (-1) to indicate that positive and negative values reflect above and below average performance. Finally, a global composite cognitive z-score was calculated by: 1) averaging the z-scores of all tests and 2) each individual's average was standardized using the mean and SD of the global mean scores.

We performed multiple linear regression models with robust covariance with cognitive tests (after z-score transformation) as the outcome variable and glaucoma as the exposure variable. All models were adjusted by the variables “previous glaucoma surgery”, “use of pilocarpine eye drops”, “use of [other] eye drop medications” (such as beta-blockers, prostaglandin analogues, and others), and “quality of vision” (dichotomized in (very) bad *vs* others, i.e., Average, Good and Very Good). Model 1 was adjusted for the sociodemographic variables age, sex, race, and education. Model 2 included the variables in Model 1 plus depression and the 9 clinical variables mentioned above. In Model 2, if significant main effects for glaucoma were observed, additional models were performed for variables that presented significant main effects to test whether there was an interaction between glaucoma and the covariate.

## Results

### Overview

Of 4331 participants, 139 (3.2%) reported glaucoma. These patients presented significantly lower rates of university degree, non-white ethnicity, and higher rates of systemic arterial hypertension. Eleven (7.9%) and 13 (9.3%) self-reported glaucoma patients reported they had undergone glaucoma eye surgery and were using pilocarpine eye drops, respectively (Table 1).

**Table 1:** Clinical and demographic characteristics of the sample (n=4,331) according to the presence of glaucoma (wave 1 - baseline - of ELSA-Brasil, 2008-2010).

Variable	No Glaucoma (n=4192)	Glaucoma (n=139)	P value
Female, n (%)	2264 (54.0%)	85 (61.2%)	0.096
Age, mean (SD)	57.5 (5.9)	58.5 (6.0)	0.051
University degree, n (%)	2429 (57.9%)	60 (43.2%)	**<0.001**
White ethnicity, n (%)	2988 (71.4%)	111 (79.9%)	**<0.001**
Good/excellent visual quality, n (%)	1198 (28.6%)	28 (20.1%)	0.029
Any depressive episode, n (%)	132 (3.1%)	6 (4.3%)	0.44
Ideal Physical Activity, n (%)	1073 (26.0%)	31 (22.8%)	0.40
Never smoker, n (%)	3479 (84.3%)	117 (86.0%)	0.59
Heavy drinker, n (%)	350 (8.4%)	13 (9.4%)	0.68
Body mass index (kg/m^2^), mean (SD)	27.3 (4.7)	27.3 (4.7)	0.95
Ideal cholesterol levels, n (%)	947 (23.0%)	36 (26.5%)	0.34
Diabetes, n (%)	954 (22.8%)	37 (26.6%)	0.29
Systemic arterial hypertension, n (%)	1758 (41.9%)	78 (56.1%)	**<0.001**
Thyroid disorders, n (%)			
Hypothyroidism	668 (15.9%)	22 (15.8%)	0.44
Hyperthyroidism	99 (2.4%)	1 (0.7%)	0.44
Self-reported heart failure, n (%)	77 (1.8%)	3 (2.2%)	0.78
Z-scores of cognitive tests			
Verbal fluency test, mean (SD)	-0.09 (1.01)	-0.33 (0.98)	**0.004**
Delayed word recall test, mean (SD)	-0.11 (1.03)	-0.19 (1.04)	0.36
Trail making test, mean (SD)	-0.16 (1.13)	-0.49 (1.45)	**0.001**
Global composite cognitive score, mean (SD)	-0.14 (1.04)	-0.38 (1.02)	0.008

For definitions of "ideal" status and estimation of z-scores, please see the main text. Between-group comparisons were performed using two-sample *t*-tests and Pearson's chi-squared tests for continuous and categorical variables, respectively. Bold type indicates significant differences (P<0.005).

### Association between cognitive performance and glaucoma

**Figure 2:**
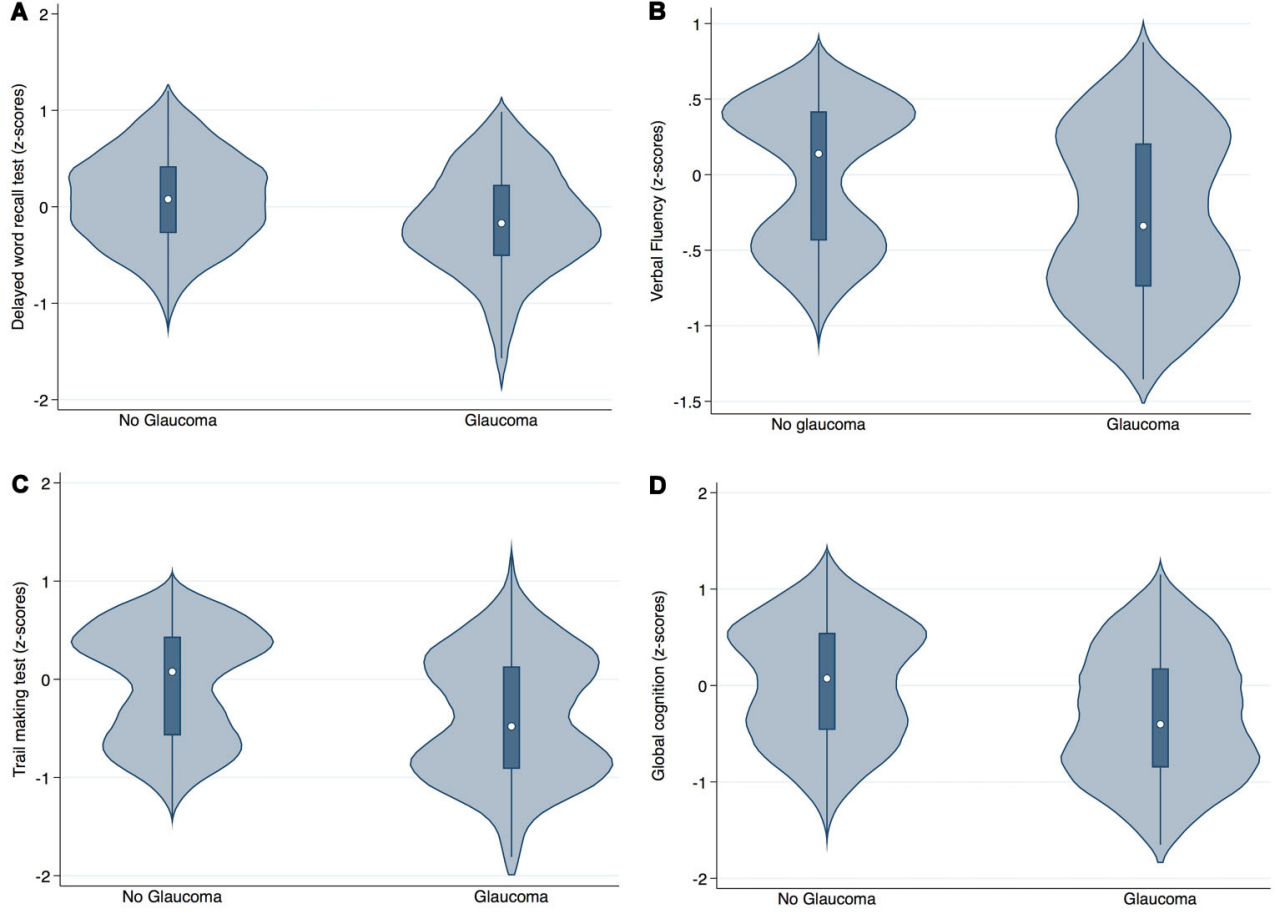
Cognitive performance in participants with and without glaucoma. Violin plots of the adjusted predicted values of z-scores of (**A**) delayed word recall test, (**B**) verbal fluency task, (**C**) trail making test, and (**D**) global cognitive composite score according to glaucoma status. Significant results were found only for verbal fluency task (**B**). Multiple linear regression models with robust covariance were employed, with adjustment for age, sex, ethnicity, education, ideal smoking status, ideal physical activity status, hypertension, diabetes, ideal cholesterol status, presence of cardiac conditions, body mass index, excessive alcohol use, presence of thyroid dysfunctions, and presence of depression.

We found a significant association between presence of glaucoma and lower performance in the phonemic verbal fluency test for both models, but not for the delayed word recall and the trail making tests. For the global composite score, there was a non-significant trend suggesting that patients with glaucoma presented lower performance (Table 2, Figure 2).

**Table 2:** Association between cognitive performance and glaucoma in the sample (n=4,331) (wave 1 - baseline - of ELSA-Brasil, 2008-2010).

	Model 1			Model 2		
	Coef (B)	95%CI	P	Coef (B)	95%CI	P
Verbal Fluency test	-0.4	-0.65 to -0.14	0.002	-0.395	-0.64 to -0.14	0.002
Delayed word recall test	0.06	-0.19 to 0.3	0.64	0.04	-0.20 to 0.29	0.72
Trail making test	-0.21	-0.61 to 0.18	0.28	-0.21	-0.61 to 0.18	0.29
Global composite cognitive score	-0.19	-0.40 to 0.03	0.09	-0.19	-0.41 to 0.02	0.08

Results from the multinomial regression models with robust covariance, according to the presence of glaucoma disease. Model 1: adjusted for age, sex, ethnicity, and education. Model 2: Model 1 + ideal smoking status, ideal physical activity status, hypertension, diabetes, ideal cholesterol status, presence of cardiac conditions, body mass index, excessive alcohol use, presence of thyroid dysfunctions, and presence of depression. CI: confidence interval.

As a significant association for verbal fluency was found, we tested interactions between glaucoma and covariates that presented main effects, which were age (P<0.001), university degree (P<0.001), white ethnicity (P<0.001), and diabetes (P<0.001). No significant interaction was found (all P*>*0.2).

## Discussion

The present study investigated the cognitive performance of participants with and without self-reported glaucoma, in a sample of 4331 participants ≥50 years of age with no history of stroke, neurocognitive disorders, cataracts, or retinal diseases, and not using drugs that interfered at cognitive functioning. We found that glaucoma patients presented poorer cognitive performance for the verbal fluency test, an association that remained significant even after adjustment for several socio-demographic and cardiovascular risk factors. For the other cognitive tests evaluated (delayed word recall and trail making test) and the global cognitive composite score, no difference in performance was observed between those with and without glaucoma.

Considering methodological aspects of our present study, the strengths were: 1) our sample was well characterized through a comprehensive assessment of clinical risk factors, use of medications, structured interview about mental disorders, and laboratory assessments; 2) we could select a specific sample of participants without major confounding variables, therefore enhancing the internal validity of our findings; 3) our sample included a diverse population regarding ethnicity and socioeconomic levels from different regions in Brazil, enhancing its generalizability; and 4) we used a stricter alpha threshold of 0.005, decreasing the chance of false positive findings.

A clear study limitation is the self-reported glaucoma diagnosis, which can result in misdiagnosis. For instance, glaucoma suspects could have been treated as glaucoma (false positives), even with no definitive confirmation in structural and functional tests. In addition, patients with asymptomatic glaucoma would be wrongly classified (false negatives). The net impact of these potential errors is unknown. Notwithstanding, the overall pattern observed in our data should still be considered, particularly considering that, if false negatives are more likely to occur (for instance, due to greater barriers to healthcare), the true trends observed in our study would be magnified, and not decreased, by this limitation.

Other study limitations include: 1) the potential for residual confounding and reverse causality (due to the cross-sectional analysis), even after adjustment for several covariates; 2) it is possible that the performance of some cognitive tasks could have been impaired due to poor vision in both groups and not glaucoma *per se*. However, this is unlikely to have occurred for the verbal fluency test that does not involve visuospatial abilities to be performed; 3) we did not assess semantic fluency; 4) we used visual acuity as a covariate adjustment in our analyses. It should be underscored that such variable is limited due to its subjective nature, and also because glaucoma leads to visual complaints only in very advanced stages, because in most cases defects initially start in the periphery of the visual fields.

Decreased phonemic verbal fluency performance was observed in glaucoma patients. This test evaluates lexical ability and executive functioning, as it requires updating abilities and inhibitory control for generating new words according to the provided instructions (20). Accordingly, the frontal and the temporal cortices are the brain regions primarily involved in phonemic and semantic verbal fluency performance, respectively (29). In addition, phonemic fluency is sensitive to subtle cognitive alterations that can indicate cognitive declining even in healthier stages (30). Thus, our findings corroborate the hypothesis that glaucoma could be a neurodegenerative disease affecting multiple cognitive domains, since glaucoma patients presented lower performance in this task. Although the etiological mechanisms were not investigated in this epidemiological study, one putative mechanism would be RGCs (a cardinal feature in glaucoma) leading to trans-synaptic anterograde degeneration in brain areas beyond the optic nerve (31). In agreement, parameters from diffusion tensor imaging showed that the fiber number connectivity in frontal and temporal areas are altered in glaucoma patients compared to healthy controls (32), corroborating our findings of poorer verbal fluency in these patients. Conversely, a recent study did not show lower verbal fluency in glaucoma patients, a finding that the authors interpreted as false-negative due to lack of power (31).

We found no decreased performance in the trail making and delayed word recall in patients with and without glaucoma. This is in contrast with a study showing decreased performance in similar tasks measuring working memory (digit span) and logic memory in those with glaucoma (31). However, differences in the tests could explain the distinct findings, since the trail making test also involves visuospatial functions in contrast with the digit span whereas logic memory requires the recall of a detailed short story and not only a list of words, being more robust to biases related to learning through repeated exposure. A recent study with 31 older adults with mild to moderate glaucoma and age-matched controls explored the performance of a computerized trail making test with different levels of complexity based on target contrast and position, observing that glaucoma patients performed poorer in these tasks, an impairment that was associated with the useful field-of-view (33). Possibly, poorer trail making performance would have been observed in our glaucoma sample if more specific tests had been employed.

In summary, we found decreased verbal fluency performance in middle-aged and older patients with glaucoma and no neurocognitive disorders. Our study is important as it suggests possible etiological links to observations that glaucoma is a neurodegenerative disease. Further longitudinal studies are necessary to better understand whether these cognitive findings precede the development of dementia, as well as to investigate whether early cognitive interventions in glaucoma patients can prevent or reverse the development of cognitive deficits.
